# The Cold-Regulated Genes of Blueberry and Their Response to Overexpression of *VcDDF1* in Several Tissues

**DOI:** 10.3390/ijms19061553

**Published:** 2018-05-23

**Authors:** Aaron Walworth, Guo-qing Song

**Affiliations:** Plant Biotechnology Resource and Outreach Center, Department of Horticulture, Michigan State University, East Lansing, MI 48824, USA; walwort8@anr.msu.edu

**Keywords:** cold-regulated genes, C-repeat-binding factor, *DDF1*, dehydration-responsive element-binding factor, freezing tolerance, tissue-specific expression, *Vaccinium corymbosum*

## Abstract

Expression of blueberry cold-regulated genes (VcCORs) could play a role in the variable cold hardiness of blueberry tissues. In this study, transcriptome comparisons were conducted to reveal expression of VcCORs in non-acclimated leaves, flower buds, and flowers of both non-transgenic and transgenic blueberries containing an overexpressed blueberry *DWARF AND DELAYED FLOWERING* gene (*VcDDF1*) as well as in fully chilled flower buds of non-transgenic blueberry. In non-transgenic blueberries, 57.5% of VcCOR genes showed differential expression in at least one of the three pairwise comparisons between non-acclimated leaves, flower buds, and flowers, and six out of nine dehydration-responsive element-binding factors showed differential expression. In addition, expression of *VcDDF1* was not cold-inducible in non-transgenic blueberries and had higher expression in flowers than in leaves or non-acclimated flower buds. In transgenic blueberries, overexpression of *VcDDF1* resulted in higher *VcDDF1* expression in leaves than in flower buds and flowers. *VcDDF1* overexpression enhanced expression of blueberry *CBF1* and *CBF3* in leaves and repressed expression of *CBF3* in both flower buds and flowers. Overall, the results revealed tissue-specific expression patterns of VcCORs. The responses of VcCORs to overexpression of *VcDDF1* suggest that it is possible to increase plant cold hardiness through overexpression of a non-cold-inducible gene.

## 1. Introduction

Deciduous fruits, such as apples, grapes, pears, stone fruits (e.g., peaches, nectarines, apricots, and cherries), and berries (e.g., blueberries, cranberries, pomegranate, and kiwifruits), are of importance for human health due to their unique nutritional and medicinal values, being packed with fiber, sugars, minerals, vitamins, and anti-oxidants. These deciduous fruit crops show a great diversity in their tolerance to extreme low/high temperatures [1,2,3]. The lowest temperature that a plant can survive is genetically controlled, but is affected by many factors, such as natural environment, acclimation, and plant developmental stage [4,5].

Climate change during the past 40 years has caused a shift toward earlier onset of the growing season of trees (e.g., 2.3 days/decade in temperate Europe) [6,7,8,9]. The early warm weather can lead to fruit/nut trees flowering out-of-season, and the increased temperature fluctuation during plant bloom often turns seasonal frost into a danger causing freezing injuries to flowers and young fruits [9,10,11]. Thus, breeding to develop freezing-tolerant fruit cultivars is considered to be a long-term solution to secure deciduous fruit production [9].

The low-temperature regulatory network in *Arabidopsis thaliana* consists of approximately 2500–4000 cold-regulated (COR) genes although tissue specificity of the expression of these COR genes has not been documented [12,13]. Of the COR genes, C-repeat-binding factor/dehydration-responsive element-binding factor 1 (CBF/DREB1) transcription factors have been well-studied due to their significant roles in tolerance to abiotic stresses, such as cold, drought, and salinity [13,14,15,16,17,18,19,20,21,22]. *CBF*/*DREB1*-mediated freezing tolerance seems to be conserved in woody fruit crops, although freezing tolerance induced by manipulation of CBF pathway genes could vary among plant species or even between tissues [18,23,24,25,26,27,28]. For example, ectopic expression of *CBF1* in strawberry increased freezing tolerance in leaves but not in receptacles [29].

Michigan ranks number one in highbush blueberry production in the United States [30,31]. The winter freezing tolerance of buds and susceptibility of buds and flowers to spring frosts are considered important genetic limitations of highbush blueberry cultivars, and the cold hardiness of blueberry buds increases during acclimation and decreases during de-acclimation and dormancy-break [32]. Constitutive expression of a highbush blueberry (*Vaccinium corymbosum*) *DWARF AND DELAYED FLOWERING 1* (*DDF1*) gene (*VcDDF1*) enhances freezing tolerance in leaves and flower buds but not in flowers [33], and transcriptome analysis has revealed that the altered expression of blueberry cold-regulated genes (VcCORs) in response to overexpression of *VcDDF1* was responsible for the increased freezing tolerance of transgenic blueberry buds [34]. In this study, transcriptome analyses were conducted to reveal tissue-specificity of the expressions of VcCORs in both non-transgenic and transgenic tissues containing *VcDDF1*. The profiles of the identified differentially expressed (DE) VcCORs in 11 comparisons are anticipated to be useful for further investigation on gene networks for cold hardiness in blueberries.

## 2. Results

### 2.1. Orthologues of Arabidopsis Cold-Regulated Genes in Blueberry

All analyses were based on high-quality RNA sequencing data of three biological replicates/plants and two technical repeats per replicate for each type of sample tested. In our blueberry transcriptome reference (GenBank accession number: SRX2728597), 24,594 transcripts of VcCORs that showed high similarities (e < −20) to 2181 *Arabidopsis* CORs (AtCORs) were annotated to 5326 unique genes using Trinotate (Table 1; Table S1). These annotated VcCORs were used for transcriptome comparisons in different tissues in this study and are useful for future studies on freezing tolerance in blueberries.

**Figure 1:**
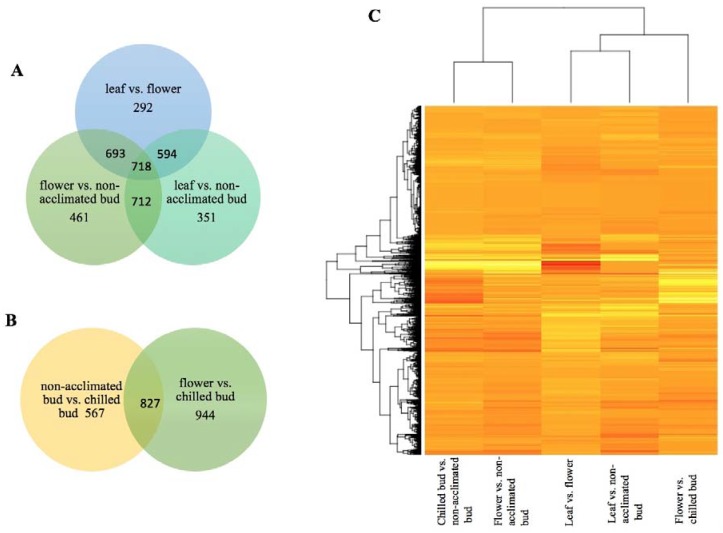
Differentially expressed (DE) orthologues of AtCORs in different tissues of non-transgenic ‘Legacy’. (**A**) Comparisons between non-acclimated leaf, bud, and flower; (**B**) A comparison between chilled and non-acclimated buds; (**C**) Heat map of the DE VcCORs in different tissues of non-transgenic ‘Legacy’. The heat map shows log_2_^Fold Change^ values for all DE VcCORs transcripts with a false discovery rate of less than 0.05.

Orthologues of approximately 50% of the AtCORs showed differential expression in each of the three transcriptome comparisons for non-acclimated blueberry tissues, including leaf versus bud, bud versus flower, and leaf versus flower (Table 1; Table S1). In total, orthologues of 1253 AtCORs showed differential expression in at least one of the three comparisons, and orthologues of 928 AtCORs did not show differential expression in any of the three comparisons. A further comparison revealed the difference between the three groups of the DE AtCOR orthologues (Figure 1A,B; Table S2).

Expressed sequencing tags (ESTs) obtained by 454 sequencing in blueberry leaves and flower buds exposed to 4 chilling hours have been generated for a major highbush blueberry ‘Bluecrop’ [35]. Using the 2181 AtCORs to search for the presence of orthologous genes in the five EST libraries, orthologues of 1169 AtCORs were detected in ‘Bluecrop’. In the five EST libraries, orthologues of 511 AtCORs were present in leaf tissues and orthologues of 553–687 AtCORs were detected in the four EST libraries for the flower buds exposed to 4 chilling hours. The non-acclimated flower buds showed more orthologues of AtCOR than the chilled flower buds (Table 1, Table S3).

### 2.2. VcCORs and Freezing Tolerance

In general, chilled blueberry flower buds are more tolerant to freezing than non-acclimated blueberry flower buds, and flowers are more vulnerable to freezing than both chilled and non-acclimated dormant buds [32]. Because of the importance of cold hardiness in dormant buds and flowers for blueberry production, DE VcCORs were identified and annotated in comparisons of chilled bud (>1000 chilling units) versus non-acclimated bud and flower versus chilled bud (Table 1; Figure 1B, C; Table S2). The chilled buds (compared to non-acclimated ones) showed DE orthologues of 953 AtCORs, and flowers (compared to chilled buds) had DE orthologues of 1131 AtCORs (Table 1). These DE VcCORs are likely responsible for the variation of freezing tolerance in these tissues.

**Figure 2:**
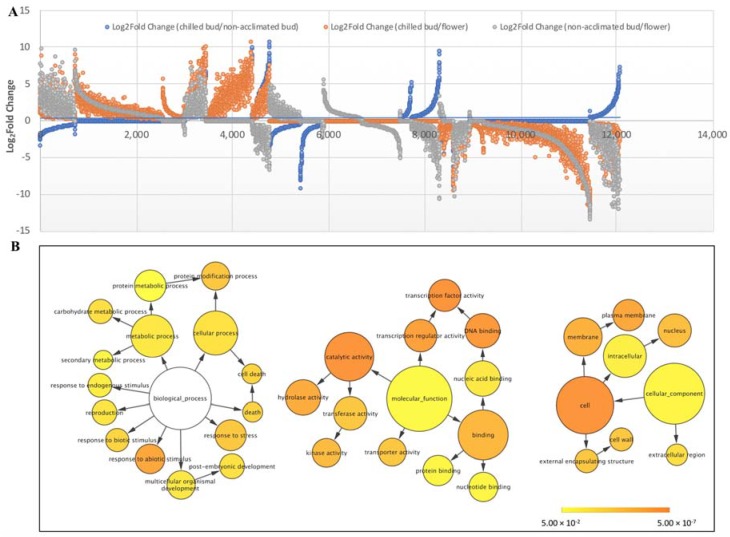
Differentially expressed (DE) VcCORs identified in transcriptome comparisons between high freezing tolerance tissues and low freezing tolerance tissues. (**A**) log_2_^Fold Change^ values for all DE VcCORs transcripts with a false discovery rate of less than 0.05; (**B**) Gene networks of the DE VcCORs transcripts in (**A**). The ontology file of GOSlim_Plants in BiNGO was used to identify overrepresented GO terms (*P* < 0.05). Bubble size and color indicate the frequency of GO term and *P*-value, respectively.

The shared DE VcCORs were identified between three groups of DE VcCORs from the comparisons of chilled bud versus non-acclimated bud, chilled bud versus flower, and non-acclimated bud versus flower. Of the shared DE VcCORs, 309 VcCORs were upregulated and 225 VcCORs were downregulated in all three groups (Figure 2A; Table S4). The gene network reveals the potential roles of these shared upregulated (309) and downregulated (225) DE VcCORs (Figure 2B; Table S4). Considering the comparison in each group was between tissues with a higher freezing tolerance and tissues with a lower freezing tolerance, these shared upregulated (309) and downregulated (225) DE VcCORs are likely responsible for the higher freezing tolerance in chilled buds (compared to non-acclimated bud and flowers) and in non-acclimated buds (compared to flowers). The *VcDDF1* (isoform # c32575_g1_i1) is among the 225 downregulated DE VcCORs (Table 2). Further analyses of the publically available EST (expressed sequence tag) data of ‘Bluecrop’ flower buds showed that *VcDDF1* (isoform # c32575_g1_i1 and c62996_g1_i1) were present only in non-acclimated flower buds in the four EST libraries for ‘Bluecrop’ flower buds with different chilling hours (Table S3). These results suggest that the *VcDDF1* is not cold inducible.

### 2.3. Expression of VcCORs in Legacy-VcDDF1-OX Plants

**Figure 3:**
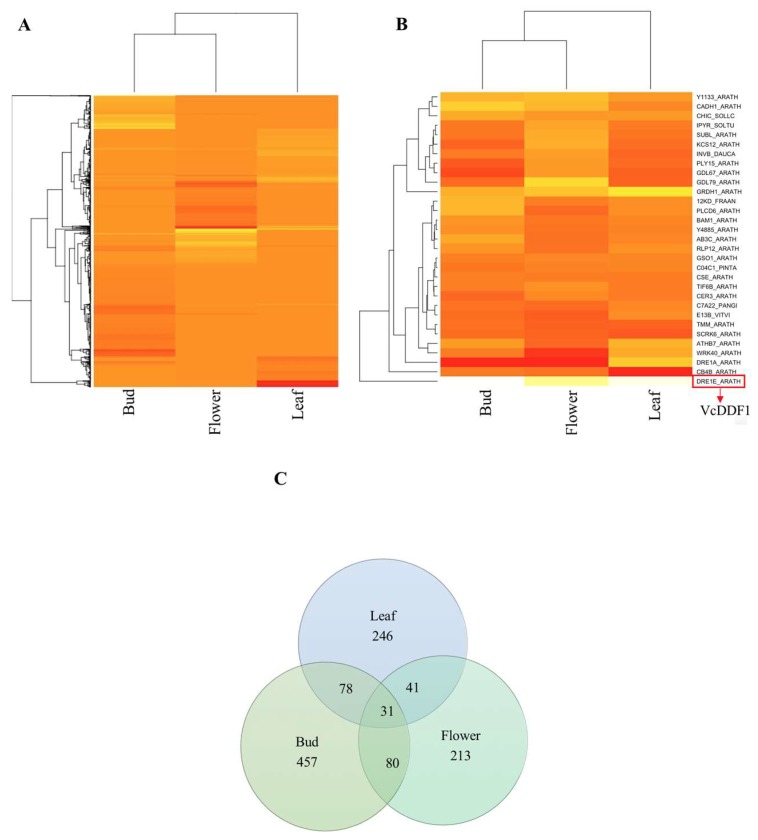
Differentially expressed (DE) VcCORs in different tissues of transgenic “Legacy-VcDDF1-OX”. (**A**) Heat map of all the DE VcCOR transcripts in the three comparisons. The heat map shows log_2_^Fold Change^ values for all DE VcCOR transcripts with a false discovery rate of less than 0.05; (**B**) Heat map of the shared DE VcCOR genes (based on annotation) in the three comparisons. The heat map shows log_2_^Fold Change^ values (means of DE transcripts) for the shared DE VcCORs genes with a false discovery rate of less than 0.05; (**C**) Comparisons of DE orthologues of AtCORs in different blueberry tissues. The numbers are the numbers of AtCORs.

*VcDDF1*-OX enhances freezing tolerance in dormant plants, leaves, and flower buds, but not significantly in flowers [33,34]. DE VcCORs, 7.4–12.1% of the total VcCORs, were identified in three tissues (leaf, non-acclimated floral bud, and flower) from the comparisons between transgenic Legacy-VcDDF1-OX and non-transgenic ‘Legacy’ (Table 1). The profiles of the DE VcCORs varied among different tissues, where the DE VcCORs in bud showed a higher similarity to those in flower than in leaf tissues (Figure 3A). DE orthologues of 38 AtCORs were identified for all three tissues; these DE orthologues were annotated to 31 unique genes, of which 3 were downregulated and 6 were upregulated in all three tissues, and fold changes of the remaining 22 genes are inconsistent in the three tissues (Figure 3B,C; Table S5).

DE VcCORs were also identified for transgenic Legacy-VcDDF1-OX plants in three transcriptome comparisons, including leaf versus bud, leaf versus flower, and flower versus bud (Table 1; Table S6). These DE VcCORs, in addition to those identified in non-transgenic ‘Legacy’ (Table 1), revealed the effect of the overexpression of *VcDDF1* on tissue-specificity of the VcCORs.

### 2.4. Blueberry CBF/DREB1 Genes

Using *CBF*/*DREB1* genes (i.e., including *CBF1-4*, *DDF1*, and *DDF2*) as queries, *VcCBF1-3* and *VcDDF1* (e <−20) were found in ‘Legacy’, which all, except *VcCBF1*, showed differential expression in the comparisons of leaf versus bud, leaf versus flower, or bud versus flower (Table 2). In addition, unlike the cold-inducible *CBFs* in *A. thaliana* [36], neither *VcCBF1-3* nor *VcDDF1* showed upregulation in chilled buds when compared to non-acclimated buds (Table 2).

In non-transgenic ‘Legacy’ plants, two *VcDDF1* homologues (c32575_g1_i1 and c62996_g1_i1) were detected. The c62996_g1_i1 did not show differential expression in the comparisons among leaf, bud, and flower tissues. Additionally, differential expression of the c62996_g1_i1 was not detected in the comparison between non-acclimated buds and chilled buds. In contrast, the c32575_g1_i1 had a higher expression in flower tissues than in both leaf and non-acclimated bud tissues and showed a lower expression in chilled buds than in non-acclimated buds (Table 2).

In transgenic Legacy-VcDDF1-OX plants, c32575_g1_i1 is the overexpressed *VcDDF1*. Compared to non-transgenic tissues of ‘Legacy’, increased expression of c32575_g1_i1 was detected in transgenic leaf (200-fold, as high as that in non-transgenic leaf), bud (154-fold), and flower (23-fold) tissues; the lower increase (23-fold) of the expression of c32575_g1_i1 in flower was due to the high c32575_g1_i1 expression in non-transgenic flower (Table 2). In the comparisons between transgenic tissues (leaf versus bud, leaf versus flower, and bud versus flower), expression of c32575_g1_i1 was 2.5-fold higher in leaf than in bud tissues, while differential expression of c32575_g1_i1 was not detected in the other two comparisons (Table 2). These results suggest that the lower increase of freezing tolerance in transgenic flowers (compared to that of leaves and buds) is not caused by the levels of *VcDDF1-*OX [33,34]*.*

In the comparisons of transgenic tissues of Legacy-VcDDF1-OX to non-transgenic tissues of ‘Legacy’, the overexpressed *VcDDF1* resulted in upregulation of *VcCBF1* and *VcCBF3* in leaf tissues and downregulation of *VcCBF3* in both bud and flower tissues (Table 2). In addition, the overexpressed *VcDDF1* upregulated the expression of the *VcDDF1* homologue c62996_g1_i1 in leaf and flower tissues but not bud tissues (Table 2).

### 2.5. Validation of the Expression of VcDDF1

**Figure 4:**
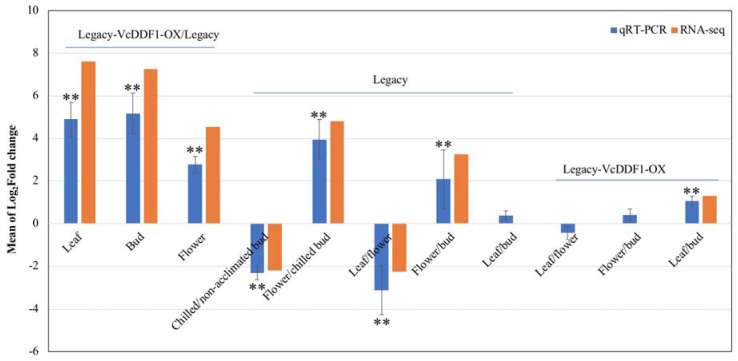
RNA sequencing and qRT-PCR analysis of *VcDDF1* (c32575_g1_i1). Eukaryotic translation initiation factor 3 subunit H is the internal control. Relative expression Log_2_^(Fold Change)^ in Legacy-VcDDF1-OX was calculated by 2^−∆∆Ct^, −∆∆Ct = −[(Ct_GOI_ − Ct_nom_)_Legacy-VcDDF1-OX_ − (Ct_GOI_ − Ct_nom_)_Legacy_]. Average fold-changes ± STDEV of three biological replicates/plants for each of Legacy-VcDDF1-OX and ‘Legacy’ plants were plotted. Significant average fold-change in qRT-PCR determined using a Student’s *t*-test is denoted. Double asterisks (**) indicate *P <* 0.01. RNA-seq data show log_2_^Fold change^ values with a false discovery rate of less than 0.05.

QRT-PCR was conducted to validate the DE or non-DE *VcDDF1* (c32575_g1_i1) in 11 transcriptome comparisons (Figure 4; Table 2). In non-transgenic ‘Legacy’, flowers showed a higher expression of *VcDDF1* than leaves and buds (chilled and non-acclimated), and non-acclimated buds had a higher *VcDDF1* expression than chilled buds, indicating that expression of *VcDDF1* is not cold-inducible. In transgenic Legacy-VcDDF1-OX plants, constitutive expression of *VcDDF1* was confirmed in all three tissues. The qRT-PCR results are consistent with the RNA-seq data (Table 2), suggesting that the result of RNA-seq data analysis in this study is reliable.

## 3. Discussion

Cold acclimation is an adaptive mechanism that has evolved to enhance cold hardiness. Functional genomics of cold acclimation is anticipated to lead to improved cold/freezing tolerance through breeding or genetic engineering.

### 3.1. VcCOR Genes in Blueberry

To date, tissue/organ specificity of the expression of COR genes in *A. thaliana* has not been well-documented. For deciduous fruit crops (e.g., blueberries), it is important to reveal the specificities of the COR orthologues because the breeding focus for improving cold hardiness is often on buds and flowers [32]. In this study, leaves and non-acclimated buds were harvested in mid-November prior to transferring the ‘Legacy’ and VcDDF-Legacy-OX plants from a heated greenhouse to natural outdoor environmental conditions. Flower and chilled bud tissues were collected from the plants after moving to natural environmental conditions. *Arabidopsis* CORs were used to identify VcCORs. The DE VcCORs in the comparison of leaf versus bud give an accurate depiction of organ-specificity of the VcCORs because the tissues were collected at the same time from the same plants. The DE VcCORs from other comparisons (i.e., leaf versus flower, chilled bud versus bud, flower versus bud, and flower versus chilled bud) revealed the difference of the expression of the VcCORs in these selected tissues (Table 1; Figure 1), although analysis of organ/tissue-specificity is confounded by the possibility of differential expression due to the effect of undocumented factors, such as the environmental conditions in which the tissues were harvested. Since the chilled buds and flowers were collected under natural growing conditions, where buds show a higher freezing tolerance than flowers and chilled buds have a higher freezing tolerance than non-acclimated buds [32], the DE VcCORs detected in the comparisons of chilled bud versus non-acclimated bud, flower versus non-acclimated bud, and flower versus chilled bud could facilitate future studies on the role of the VcCORs in the cold hardiness of blueberries.

### 3.2. VcDDF1 and Freezing Tolerance

The AP2/ERFs play a significant role in plant responses to several abiotic stresses (e.g., cold, dehydration, and high salinity), which has stimulated many recent studies on genome-wide analysis of the AP2/ERF in several plant species [17]. The CBF/DREB1 genes belong to a large family of the AP2/ERF transcription factors and have a conserved DNA binding domain recognizing the dehydration-responsive element/C-repeat (DRE/CRT) *cis*-acting element in the promoters of their target genes [17,19,36]. This CBF/DREB1 pathway has been well-documented in *A. thaliana* [19,20,21,22]. It appears that most of the plant species tested undergo cold acclimation through a universal process that belongs at least partially to the CBF/DREB1-mediated cold-response pathway [15,16,17,18,37].

As revealed in *A. thaliana*, the low-temperature regulatory network is more complicated than the CBF-CRT/DRE regulatory module [12,13,38]. For example, acclimation (cold) induces differential expression of 2637 cold-regulated (AtCOR) genes. In contrast, overexpression of *CBF1*, *CBF2*, and *CBF3* can only alter 171 (6.5%) AtCOR genes. For deciduous fruit crops, many efforts have been focusing mainly on developing transgenic plants for freezing tolerance using *CBF* or *CBF* orthologues. The CBF-CRT/DRE regulatory module has not been well-studied through forward genetics or reverse genetics. In our recent studies, transcriptomic responses to overexpression of the *VcDDF1* showed DE COR genes (compared to non-transgenic plants) that may contribute to an increase in freezing tolerance [34].

To date, no profile of DE VcCORs between different blueberry organs has been documented. ESTs in blueberry leaves and flower buds exposed to different chilling hours have been generated through 454 sequencing for a major highbush blueberry ‘Bluecrop’ [35]. In this study, profiles of DE VcCORs were developed for 11 comparisons (Table 1, Figure 1). Given that chilled/acclimated flower buds have a higher freezing tolerance than non-acclimated buds and non-acclimated buds show a higher freezing tolerance than flowers in blueberries [32,33], the DE CORs revealed in comparisons of the three groups (i.e., chilled bud versus non-acclimated bud, non-acclimated bud versus flower, and chilled bud versus flower) of DE COR genes facilitate our understanding of the potential COR genes that can be used to increase freezing tolerance in buds and flowers through manipulating those shared upregulated (309) and downregulated DE orthologues of 309 and 225 AtCORs, respectively (Figure 2).

*DDF1* is not cold inducible in *A. thaliana* [12]. Similarly, the *VcDDF1* is not cold inducible. This was supported both by our RNA-seq data and the EST data in the four EST libraries for ‘Bluecrop’ flower buds with different chilling hours (Table 2; Table S3). It is interesting that the non-cold-inducible *VcDDF1* functions as a promoter of freezing tolerance. In non-transgenic ‘Legacy’, flowers show a higher expression of *VcDDF1* than both leaves and buds (Table 2); physiologically, this seems to be consistent with our previous observation where flowers exhibited a lower EL_50_ (temperature at which 50% of maximum electrolyte leakage occurred) [33]. In addition, the overexpressed *VcDDF1* resulted in increased freezing tolerance in transgenic VcDDF-Legacy-OX plants [34]. Transcriptome comparisons between transgenic and non-transgenic tissues (leaf, bud, and flower) revealed that the overexpressed *VcDDF1* changed the expression profile of the VcCORs (Figure 3), which could be responsible for the increased freezing tolerance. Unlike overexpression of cold-inducible CBFs, which often results in a trade-off impact between cold tolerance and plant growth in *A. thaliana* [13], overexpression of the cold-repressive *VcDDF1* increased freezing tolerance without obvious impact on plant growth [34].

## 4. Method

### 4.1. Plant Materials

Non-transgenic southern highbush blueberry ‘Legacy’ and a representative transgenic ‘Legacy’ (hereafter Legacy-VcDDF1-OX, previously named as II7) was used in this study.

Legacy-VcDDF1-OX contains a blueberry-derived CBF gene (AVI45245.1), which was designated as *BB-CBF* [33,39] and renamed as *VcDDF1* [34]. Production and phenotypic analysis of transgenic ‘Legacy’ events containing a CaMV 35S promoter-driven *VcDDF1* were described in our previous report [33].

Twelve plants for each of non-transgenic and transgenic ‘Legacy’ were obtained through micropropagation of in vitro cultured shoots. All plants unless otherwise mentioned were grown normally and were fully chilled (>1200 chilling unit) in winter in a secured courtyard under natural light conditions at Michigan State University, East Lansing, Michigan (latitude 42.701847, longitude −84.482170). The average low and high temperatures in January are −10.6 °C and −1.8 °C, respectively (Available online: http://www.usclimatedata.com/climate/east-lansing/michigan/united-states/usmi0248).

Non-acclimated flower buds (30–50 buds per plant) and leaves (2–3 g per plant) were collected in November from three plants for each of the non-transgenic ‘Legacy’ and transgenic Legacy-VcDDF1-OX before the plants were exposed to chilling treatments. Chilled flower buds (30–50 buds per plant) of ‘Legacy’ were from three plants grown in the courtyard with natural environmental conditions and were harvested at the end of January. Late-pink buds, 20–30 buds per plant, were harvested in April from three ‘Legacy’ and three Legacy-VcDDF1-OX plants grown in the courtyard. All tissues collected were frozen immediately in liquid nitrogen and stored at −80 °C. Three biological replicates, defined as tissues from three plants, were used for transcriptome and quantitative RT-PCR (qRT-PCR) analysis [34].

### 4.2. RNA Preparation, Sequencing, and de Novo Transcriptome Assembly

Total RNA isolation, RNA sequencing using the Illumina HiSeq2500 platform, and de novo transcriptome assembly using the Trinity platform (trinity/20140413p1) [40] were described in our recent report [41].

### 4.3. Differential Expression Analysis and Transcriptome Annotation

RNA-seq reads of three biological replicates/plants and two technical replicates for each biological replicate were analyzed. These RNA-seq reads are available in Genbank (SRA accession: SRP103678). The paired reads were aligned to the transcriptome reference developed for ‘Legacy’ [41] and the abundance of each read was estimated using the Trinity command “align_and_estimate_abundance.pl”. The Trinity command “run_DE_analysis.pl –method edgeR” was used for differential expression analysis. The DE genes or transcripts (relative to non-transgenic ‘Legacy’ unless other mentioned) with false discovery rate (FDR) values below 0.05 were used for further analyses [34]. Transcriptome annotation was performed using Trinotate_v2.0 (Available online: https://trinotate.github.io).

### 4.4. Expressed Sequence Tags (ESTs) in Blueberry

The ESTs of ‘Bluecrop’ leaves and flower buds at four different stages of cold acclimation were downloaded from the Blueberry Genomics Database website (Available online: http://bioinformatics.towson.edu/BBGD454/). The retrieved sequences were used for VcCOR analysis using the tblastn command of BLAST+.

### 4.5. Identification of the VcCORs

The 2637 cold-regulated genes (CORs) identified in wild-type *A. thaliana* plants and 172 CORs differentially expressed at a warm temperature (22 °C) in transgenic *A. thaliana* plants overexpressing *CBF1*, *CBF2*, or *CBF3* were obtained from Park et al. [12]. These CORs were used to identify their orthologues in blueberry (VcCORs).

Representative protein sequences of selected genes of *A. thaliana* were download from the TAIR server (Available online: https://www.arabidopsis.org/tools/bulk/sequences/index.jsp). The retrieved sequences were used to search for the transcriptome reference of blueberry (GenBank accession number: SRX2728597) using the tblastn command of BLAST+. The resultant transcripts that show e-values lower than −20 were used to screen the retrieved ‘Bluecrop’ EST sequences and the DE transcript list of each comparison.

### 4.6. QRT-PCR Analysis

The RNA samples used for RNA-sequencing were used for cDNA preparation. Reverse transcription of RNA to cDNA was performed using SuperScript II reverse transcriptase (Invitrogen, Carlsbad, CA, USA). The resulting cDNA of one microgram of RNA was diluted (volume 1: 4) in water and 1 μL/sample (25 ng) was used for PCR reactions. PCR primers cbF3: 5′-ATTGGCGCTGAAAAGTGAGT-3′ and cbR1: 5′-CGCTGCCCTCCTTATATGTT-3′ that cover a 109-bp region of the VcDDF1 were used. Primers of eukaryotic translation initiation factor 3 subunit H (VcEIF-F: 5′-GAGAGATTCAGATGCCCAGAAG-3′ and VcEIF-R: GGACAATGGATGGACCAGATT) were the internal control. QRT-PCR was performed in triplicate (three biological controls) on an Agilent Technologies Stratagene Mx3005P (Agilent Technologies, Santa Clara, CA, USA) using the SYBR Green system (Life Technologies, Carlsbad, CA, USA). In each 25 µL reaction mixture, 25 ng cDNA, 200 nM primers, and 12.5 µL of 2× SYBR Green master mix were included. The reaction conditions for all primer pairs were 95 °C for 10 min, 40 cycles of 30 s at 95 °C, 60 s at 60 °C, and 60 s at 72 °C followed by one cycle of 60 s at 95 °C, 30 s at 55 °C, and 30 s at 95 °C. The specificity of the amplification reaction for each primer pair was determined by the melting curve. Transcript levels within samples were normalized to the eukaryotic translation initiation factor 3 subunit H. Log_2_^(Fold Change)^ was calculated using –∆∆Ct = −[(Ct_GOI_ − Ct_nom_)_Mu_-_Legacy_ − (Ct_GOI_ − Ct_nom_)_Legacy_] for each transgenic Mu-Legacy versus a non-transgenic ‘Legacy’ sample (*n* = 3) [42].

### 4.7. Gene Network Construction

Annotated transcripts were imported to Cytoscape 3.5.0 under BiNGO’s default parameters with the selected ontology file ‘GOSlim_Plants’ and selected organism *A. thaliana* [43,44].

## 5. Conclusions

The DE VcCORs identified in the comparisons of transcriptomes of eight tissues revealed the specificity of the VcCORs expression in different tissues and different chilling stages. *VcDDF1* was not cold inducible; however, overexpression of the *VcDDF1* increased freezing tolerance through its impact on the other DE VcCORs. The results of this study will facilitate future studies on improving blueberry cold hardiness through manipulating VcCORs.

## Figures and Tables

**Table 1:** Summary of transcriptome comparisons used to identify differentially expressed (DE) orthologues of AtCORs in different blueberry tissues.

Source of Transcripts	Transcriptome Comparison	Percentage (Number) of AtCORs that Have DE Blueberry Orthologues)	Percentage (Number) of Unique Genes out of the Annotation of the DE VcCORs ** (Annotated by Trinotate)	Total Number of AtCORs that Have (DE) Blueberry Orthologues)	Total Number of Unique Genes out of the Annotation of the (DE) VcCORs (Annotated by Trinotate)
Blueberry transcriptome reference: reftrinity				2181	5326
Bluecrop leaf (MID5) *				511	685
Bluecrop bud (MID10: 0 CU) *				687	996
Bluecrop bud (MID4: 397 CU) *				620	874
Bluecrop bud (MID1: 789 CU) *				610	873
Bluecrop bud (MID2: 1333 CU) *				553	802
Bluecrop leaf and bud (MID5, MID10, MID4, MID1, and MID2) *				1169	1960
Legacy	Leaf versus flower	49.2% (1074/2181)	58.7% (3126/5326)	(1074)	(3126)
	Flower versus non-acclimated bud	52.9% (1154/2181)	69.1% (3678/5326)	(1154)	(3678)
	Leaf versus non-acclimated bud	50.8% (1108/2181)	61.8% (3293/5326)	(1108)	(3293)
	Flower versus chilled bud	51.9% (1131/2181)	64.5% (3434/5326)	(1131)	(3434)
	Chilled bud versus non-acclimated bud	43.7% (953/2181)	49.5% (2639/5326)	(953)	(2639)
Legacy-VcDDF1-OX	Leaf versus flower	49.9% (1089/2181)	59.7% (3178/5326)	1089	(3178)
	Flower versus non-acclimated bud	52.7% (1150/2181)	67.7% (3606/5326)	1150	(3606)
	Leaf versus non-acclimated bud	53.0% (1156/2181)	66.5% (3542/5326)	1156	(3542)
Legacy and Legacy-VcDDF1-OX	Legacy-VcDDF1-OX leaf versus Legacy leaf	12.9% (282/2181)	7.4% (396/5326)	282	(396)
	Legacy-VcDDF1-OX flower versus Legacy flower	11.4% (248/2181)	6.9% (365/5326)	248	(365)
	Non-acclimated Legacy-VcDDF1-OX bud versus non-acclimated Legacy bud	17.8% (389/2181)	12.1% (646/5326)	389	(646)

* 454 expressed sequencing tag (EST) sequencing data (Available online: http://bioinformatics.towson.edu/BBGD454/); ** blueberry cold-regulated genes (VcCORs): orthologues (e <−20) of 2181AtCORs.

**Table 2:** Differential expression of CBF/DREB1 and DREB2 transcription factors in different comparisons.

Transcript	Arabidopsis1	Transcription Factors	E-Value	Annotation	Log_2_^Fold Change^ (Legacy-VcDDF1-OX/Legacy)			Log_2_^Fold Change^ for Legacy						Log_2_^Fold Change^ for Legacy-VcDDF1-OX		
					**Leaf**	**Non-Acclimated Buds**	**Flower**	**Chill Bud/Non-Acclimated Bud**	**Flower/Chilled Buds**	**Leaf/Flower**	**Flower/Non-Acclimated Bud**	**Leaf/Non-Acclimated Bud**	**Specificity**	**Leaf/Flower**	**Flower/Non-Acclimated Bud**	**Leaf/Non-Acclimated Bud**
c88132_g2_i2	AT4G25490	CBF1	2.00 × 10^−21^	DRE1B_ARATH	3.21	#N/A	#N/A	#N/A	#N/A	#N/A	#N/A	#N/A	leaf = bud = flower	3.70	#N/A	3.93
c75369_g2_i1	AT4G25470	CBF2	3.00 × 10^−21^	ERF38_ARATH	#N/A	#N/A	#N/A	#N/A	#N/A	#N/A	#N/A	#N/A	leaf = bud = flower	3.08	#N/A	1.76
c85919_g2_i1	AT4G25470	CBF2, CBF1, CBF4, CBF3, DDF1	5.00 × 10^−45^	DRE1F_ORYSJ	#N/A	#N/A	#N/A	#N/A	4.50	−1.88	2.67	#N/A	leaf = bud < flower	#N/A	#N/A	#N/A
c85919_g2_i2	AT4G25470	CBF2, CBF1, CBF4, CBF3, DDF1	7.00 × 10^−45^	DRE1F_ORYSJ	#N/A	#N/A	#N/A	#N/A	3.10	−1.87	2.05	#N/A	leaf = bud < flower	#N/A	#N/A	#N/A
c85919_g2_i4	AT4G25470	CBF2, CBF1, CBF4, CBF3, DDF1	3.00 × 10^−45^	DRE1F_ORYSJ	#N/A	#N/A	#N/A	−2.20	5.80	−2.48	4.35	#N/A	leaf = bud < flower	#N/A	4.04	4.03
c85919_g2_i5	AT4G25470	CBF2, CBF1, CBF4, CBF3, DDF1	6.00 × 10^−45^	DRE1F_ORYSJ	#N/A	#N/A	#N/A	#N/A	4.50	−2.30	5.45	#N/A	leaf = bud < flower	#N/A	4.23	4.84
c85919_g2_i6	AT4G25470	CBF2, CBF1, CBF4, CBF3, DDF1	3.00 × 10^−45^	DRE1F_ORYSJ	#N/A	#N/A	#N/A	#N/A	5.10	−3.42	6.06	#N/A	leaf = bud < flower	#N/A	4.97	4.42
c82156_g1_i1	AT4G25470	CBF2, DDF1	1.00 × 10^−22^	ERF23_ARATH	#N/A	#N/A	#N/A	#N/A	−3.90	6.35	−4.69	1.67	flower < bud < leaf	5.32	−2.57	2.78
c91057_g4_i1	AT4G25470	CBF2, DDF1, CBF1	2.00 × 10^−26^	ERF43_ARATH	#N/A	#N/A	#N/A	−1.80	−5.30	#N/A	−7.56	−6.37	leaf = flower < bud	#N/A	−9.70	−8.40
c97417_g2_i1	AT4G25470	CBF2, DDF1, CBF1, CBF3, CBF4	2.00 × 10^−27^	TINY_ARATH	#N/A	#N/A	−0.65	#N/A	3.10	−1.24	2.63	1.37	bud < leaf < flower	−1.57	2.69	1.13
c88132_g2_i1	AT4G25480	CBF3, CBF2, CBF1, CBF4, DDF1	2.00 × 10^−61^	DRE1A_ARATH	2.47	−1.87	−2.96	#N/A	2.90	−2.63	#N/A	−2.06	leaf < bud = flower	2.78	#N/A	2.21
c77615_g1_i1	AT1G12610	DDF1, CBF2	4.00 × 10^−27^	DREB3_ARATH	#N/A	#N/A	#N/A	#N/A	−1.00	2.58	−1.61	#N/A	flower < leaf = bud	#N/A	#N/A	#N/A
c87707_g1_i1	AT1G12610	DDF1, CBF2	5.00 × 10^−24^	DREB3_ARATH	#N/A	#N/A	#N/A	#N/A	−0.90	1.51	−1.16	#N/A	flower < leaf = bud	1.46	−1.20	#N/A
c91057_g4_i3	AT1G12610	DDF1, CBF2	2.00 × 10^−23^	DREB3_ARATH	#N/A	#N/A	#N/A	−1.00	−7.50	3.96	−8.75	−4.81	Flower < leaf < bud	2.69	−8.23	−5.54
c32575_g1_i1	AT1G12610	DDF1, CBF2, CFB3, CBF1, CBF4	2.00 × 10^−42^	DRE1E_ARATH	7.64	7.27	4.54	−2.20	4.80	−2.26	3.25	#N/A	leaf = bud < flower	#N/A	#N/A	1.31
c62996_g1_i1	AT1G12610	DDF1, CBF2, CFB3, CBF1, CBF4	5.00 × 10^−44^	DRE1E_ARATH	4.61	#N/A	3.96	#N/A	#N/A	#N/A	#N/A	#N/A	leaf = bud = flower	#N/A	#N/A	#N/A

#N/A: No differential expression.

## Supplementary Materials

Supplementary materials can be found at http://www.mdpi.com/1422-0067/19/6/1553/s1.
